# P-632. Evaluation of the BIOFIRE® SPOTFIRE® Respiratory/Sore Throat (R/ST) Panel Mini for Use With Anterior Nasal Swab Specimens in the Near-Patient Testing Setting

**DOI:** 10.1093/ofid/ofaf695.845

**Published:** 2026-01-11

**Authors:** Jared Goos, Ashley Ky, Kristen Holmberg, Christopher Morgan, Daria Drobysheva, Samantha King, Rangaraj Selvarangan, Dinah Dosdos, Mark Steele, Amy M Stubbs, Daniel M Cohen, Thomas Davis, Jennifer Dien Bard, Cristina Costales, Deborah R Liu, Cynthia Andjelic

**Affiliations:** bioMerieux, Salt Lake City, UT; bioMérieux, Salt Lake City, Utah; bioMerieux, Salt Lake City, UT; bioMerieux, Salt Lake City, UT; bioMerieux, Salt Lake City, UT; bioMerieux, Salt Lake City, UT; Children’s Mercy Hospital, Kansas City, Missouri; Children's Mercy Hospital Kansas City, Kansas City, Missouri; University Health Truman Medical Center, Kansas City, Missouri; University Health-TMC/University of Missouri-Kansas City, FAIRWAY, Kansas; Nationwide Children's Hospital, Columbus, Ohio; Indiana university, Indianapolis, Indiana; Children's Hospital Los Angeles; University of Southern California, Los Angeles, CA; Children's Hospital Los Angeles, Los Angeles, California; Children's Hospital Los Angeles, Los Angeles, California; bioMerieux, Salt Lake City, UT

## Abstract

**Background:**

The BIOFIRE^®^ SPOTFIRE^®^ Respiratory/Sore Throat (R/ST) Panel Mini provides results for five viral pathogens commonly responsible for upper respiratory tract infection (Respiratory menu) or one bacterial and four viral pathogens commonly responsible for pharyngitis (Sore Throat menu). For the Respiratory menu, the test is currently indicated for use with nasopharyngeal swab (NPS) specimens in viral transport media. NPS specimens may cause discomfort, which can lead to inconsistent sample quality or refusal to provide a specimen, especially in children. Anterior nasal swab (ANS) specimens are a common specimen type for many diagnostic tests and are more convenient and comfortable to collect. The purpose of this study was to assess the performance of the BIOFIRE SPOTFIRE R/ST Panel Mini when testing ANS specimens in viral transport media.

**Methods:**

ANS specimens were collected from consented/assented subjects with signs and symptoms of upper respiratory tract infection in near-patient testing settings. Enrollment was conducted between March 2024 and February 2025 at five study sites in the US. Each ANS specimen was tested at the study sites using the BIOFIRE SPOTFIRE R/ST Panel Mini by non-laboratory healthcare professionals. Performance of the BIOFIRE SPOTFIRE R/ST Panel Mini when using ANS specimens was determined by a comparison to FDA-cleared molecular tests.

**Results:**

A total of 805 ANS specimens were collected and tested with the BIOFIRE SPOTFIRE R/ST Panel Mini. When testing ANS specimens, the BIOFIRE SPOTFIRE R/ST Panel Mini demonstrated an overall positive percent agreement (PPA) of 95.4% and a negative percent agreement (NPA) of 99.1% in comparison to the reference methods.

**Conclusion:**

The BIOFIRE SPOTFIRE R/ST Panel Mini is a sensitive, specific, and robust test for the rapid detection of five common viral pathogens responsible for upper respiratory tract infection when using ANS specimens in a near-patient testing setting. The convenience, comfort, and patient acceptance of ANS specimens is expected to aid in the effective diagnosis and management of upper respiratory tract infections in near-patient testing settings.

The BIOFIRE SPOTFIRE R/ST Panel Mini is not cleared or approved for use with ANS specimens in viral transport media.

**Disclosures:**

Jared Goos, PhD, bioMerieux: Employment Ashley Ky, MLS(ASCP), bioMerieux: Employment Kristen Holmberg, n/a, bioMerieux: Employment Christopher Morgan, n/a, bioMerieux: Employment Daria Drobysheva, PhD, bioMerieux: Employment Samantha King, n/a, bioMerieux: Employment Rangaraj Selvarangan, PhD, Altona: Grant/Research Support|Biomerieux: Advisor/Consultant|Biomerieux: Grant/Research Support|Biomerieux: Honoraria|Cepheid: Grant/Research Support|Hologic: Grant/Research Support|Hologic: Honoraria|Meridian: Grant/Research Support|Qiagen: Grant/Research Support Mark Steele, MD, BioFire Diagnostics, LLC: Grant/Research Support|T2 Biosystems, Inc.: Grant/Research Support Amy M. Stubbs, MD, bioMerieux/BioFire Diagnostics: Grant/Research Support|T2 Biosystems: Grant/Research Support Jennifer Dien Bard, PhD, D(ABMM), FIDSA, bioMerieux: Advisor/Consultant|bioMerieux: Grant/Research Support Cristina Costales, MD, Amgen Inc.: Stocks/Bonds (Private Company)|Biomerieux: Grant/Research Support Cynthia Andjelic, PhD, bioMerieux: Employment

